# Dynamic control of lithium dendrite growth with sequential guiding and limiting in all-solid-state batteries

**DOI:** 10.1126/sciadv.adw9590

**Published:** 2025-08-20

**Authors:** Longbang Di, Zongji Huang, Lei Gao, Yunxing Zuo, Jinlong Zhu, Mengyu Sun, Shusen Zhao, Jiaxin Zheng, Songbai Han, Ruqiang Zou

**Affiliations:** ^1^ School of Advanced Materials, Peking University, Shenzhen Graduate School, Shenzhen 518055, China.; ^2^ Shenzhen Key Laboratory of Solid State Batteries, Southern University of Science and Technology, Shenzhen 518055, China.; ^3^ EACOMP, Shenzhen 518055, China.; ^4^ Department of Physics, Southern University of Science and Technology, Shenzhen 518055, China.; ^5^ National Center for Applied Mathematics Shenzhen (NCAMS), Shenzhen 518055, China.; ^6^ State Key Laboratory of Advanced Waterproof Materials, School of Materials Science and Engineering, Peking University, Beijing 100871, China.

## Abstract

Solid-state electrolyte (SSE) is anticipated to exhibit proper mechanical strength and effectively inhibit the penetration of lithium dendrites. However, the growth of lithium dendrites is inevitable, driven by the intrinsic properties of SSEs. Hence, guiding the growth of lithium dendrites in a controllable way is more feasible instead of completely preventing their growth. Here, we present a strategically designed structural layer composed of graded lithium nitride particles, which guides the growth of lithium dendrites within confined spaces. Meanwhile, this layer is paired with a less lithium-stable electrolyte and enables the guided lithium dendrites to self-limit within localized regions at the interface. The comprehensive analysis further reveals that the designed bilayer SSE effectively harnesses the interface-generated pressure during battery cycling, achieving dynamic control of lithium dendrite growth. This interfacial structure design of SSE holds broad applicability for regulating lithium dendrites in all-solid-state lithium-metal batteries.

## Teaser

A designed electrolyte structure controls lithium dendrites and enhances stability of all-solid-state lithium metal batteries.

## INTRODUCTION

All-solid-state batteries (ASSBs), using solid-state electrolytes (SSEs) and a Li metal anode, offer a viable solution for addressing the safety and specific capacity apprehensions inherent in traditional lithium-ion batteries (*1*, *2*). Nevertheless, there are still challenges pertaining to an effective pairing of SSEs with Li metal anode in ASSBs, considering the practical requirements (*3*–*8*).

Among the array of interface compatibility challenges encountered between SSEs and Li metal, the growth of Li dendrites has posed a formidable obstacle. In the initial stages of proposing the concept of ASSBs, SSEs were anticipated to prevent the formation of Li dendrites owing to their higher shear modulus than liquid electrolytes. Representatively, the kinetic model for dendrite propagation, as proposed by Monroe and Newman (*9*), suggests that SSEs with a shear modulus twice as high as that of Li metal (~3 GPa) can effectively inhibit the growth of Li dendrites. However, even in garnet-type oxide Li_7_La_3_Zr_2_O_12_ with ultrahigh shear modulus (51 to 62 GPa) (*10*), the growth and propagation of Li dendrites can be extensively observed at a lower current and capacity relative to the liquid electrolytes (*11*–*13*). It has been demonstrated that initiation and propagation of Li dendrites in SSEs are driven by the intrinsic characteristics of polycrystalline SSEs such as grain boundaries (GBs), cracks, and pores (*14*).

Numerous studies have been conducted with the goal of optimizing GBs and minimizing porosity of SSEs to mitigate the issue of Li dendrite growth (*15*–*21*). However, even with intricate interface engineering, intrinsic inhomogeneities, GBs, and defects within polycrystalline SSEs persist, limiting the suppression of Li dendrite growth and making its complete elimination unattainable. Therefore, to tackle the issue of Li dendrites, it is essential to adopt strategies that go beyond traditional methods of aiming at suppressing their growth.

**Fig. 1.**
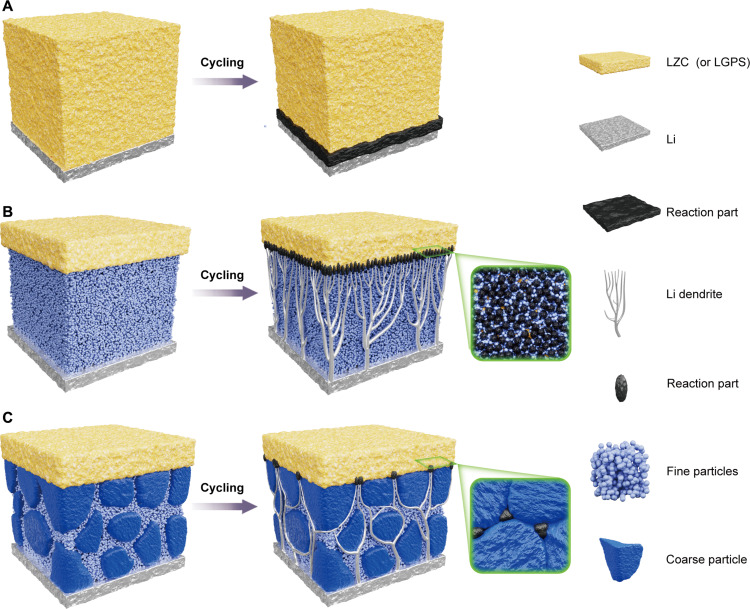
Schematic diagram of the structural design for SSEs. (**A**) Li|LZC (or LGPS). LZC (or LGPS) reacts directly with Li metal, causing interface failure. (**B**) Li|Li_3_N|LZC (or LGPS). The excessive Li dendrites penetrate the Li_3_N layer composed of fine particles, which leads to deterioration of the interface between LZC (or LGPS) and Li_3_N. (**C**) Li|Li_3_N|LZC (or LGPS). The growth of Li dendrites is guided by Li_3_N layer composed of coarse and fine particles, which leads to the self-limiting behaviors of Li dendrites, effectively improving the stability of interface.

In this work, a composite bilayer electrolyte structure was developed, featuring a less Li-stable layer and a Li-stable graded particle layer. This innovative design addresses the challenge of Li dendrites by first guiding them into confined localized regions and then limiting their growth (Fig. 1). Specifically, the Li-stable graded particle layer consists of coarse Li_3_N particles and fine Li_3_N particles, which guides the distribution of Li dendrites to bypass the coarse particles while enabling their growth in the localized regions of the fine particles. The coarse particles act as mechanical supports, restricting dendrite growth, while the fine particles fill the gaps, ensuring proper interface contact and enabling controlled propagation of Li dendrites. In addition, the chemical/electrochemical stability of Li_3_N with Li metal, coupled with its high ionic conductivity, ensures both interfacial stability and efficient Li^+^ transport. On the other side, the confined spreading Li dendrites react with less Li-stable electrolyte layer, such as Li_2_ZrCl_6_ (LZC) or Li_10_GeP_2_S_12_ (LGPS) (*22*), and undergo self-limitation under stress between Li dendrites and LZC (or LGPS). This interface design concept, which guides rather than completely suppresses Li dendrite growth, aligns with the nature of Li dendrites and controllably regulates their growth within the SSEs.

## RESULTS

### Electrochemical performance of the batteries

**Fig. 2.**
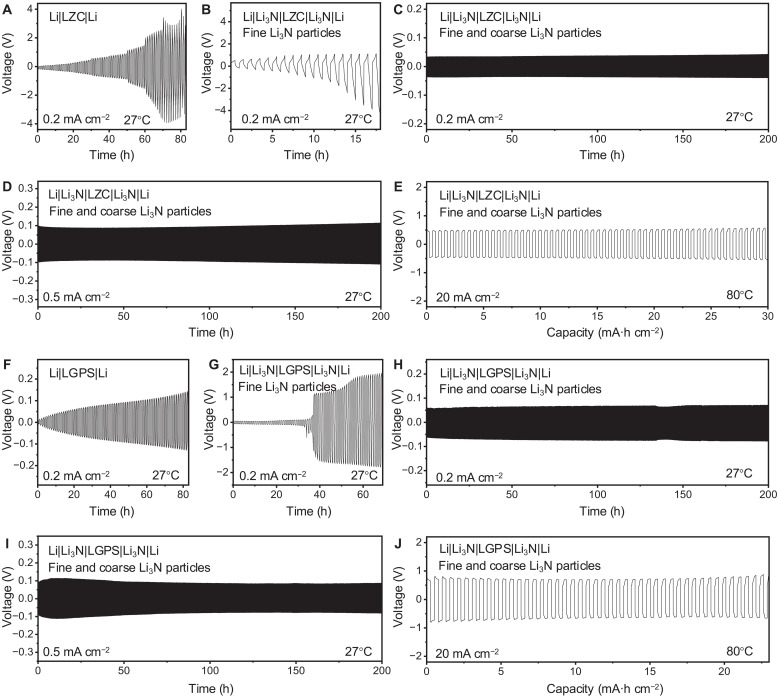
The performance of Li|SSE|Li cells. (**A**) Symmetric Li cell with LZC as SSE, cycling at 0.2 mA cm^−2^ at 27°C for 1 hour (h) in each cycle. (**B** to **E**) The Li|Li_3_N|LZC|Li_3_N|Li symmetric cells, cycling at 0.2, 0.5, and 20 mA cm^−2^. (**F**) Symmetric Li cell with LGPS as SSE, cycling at 0.2 mA cm^−2^ at 27°C for 1 hour in each cycle. (**G** to **J**) The Li|Li_3_N|LGPS|Li_3_N|Li symmetric cells, cycling at 0.2, 0.5, and 20 mA cm^−2^. The Li_3_N layers in (B) and (G) are composed of fine Li_3_N particles, whereas those in (C) to (E) and (H) to (J) consist of both fine and coarse Li_3_N particles.

Halide SSEs typically exhibit excellent ionic conductivity (fig. S1), sometimes even comparable to that of liquid electrolytes (*23*). However, the undesirable compatibility with Li metal hinders their application in high–energy density ASSBs equipped with Li metal anode. As presented in Fig. 2A and figs. S2, S3A, and S4, symmetric Li metal cells and ASSBs using halide LZC electrolyte exhibit rapid degradation, which can be attributed to the continuous reduction of cations by Li metal (Fig. 1A) (*24*). Inspired by the concept of the interface design proposed by Li *et al.* (*22*), if the less Li-stable SSE is sandwiched between Li-stable SSEs, then this configuration can block the continuous reaction at the Li|SSE interface and attain dynamic stability. Unfortunately, as illustrated in Fig. 2B and fig. S5, both the symmetric Li metal cell and ASSBs using LZC coupled with Li_3_N layers composed of uniformly sized fine particles display unstable performance. Furthermore, we disassembled the symmetric Li metal cell to observe the morphology of the Li_3_N|LZC interface after galvanostatic cycling. As demonstrated in fig. S6, the surfaces of LZC undergo deterioration with the formation of by-products, even when protected by Li_3_N layers. This anomalous phenomenon can be ascribed to an interface mechanism, where excessive Li dendrites penetrate the Li_3_N layer, accumulating and reacting with LZC electrolyte, leading to failure at the Li_3_N|LZC interface (fig. S7). That is, although chemical reactions between LZC and Li dendrites can shut off further growth of the dendrites, this comes at the expense of extensive surface failure of LZC (Fig. 1B).

To mitigate failure at the Li_3_N|LZC interface, we strategically designed the graded Li_3_N particle layer, consisting of coarse particles and fine particles, to guide Li dendrites to spread within localized regions. Considering the thickness of the Li_3_N layer and the performance requirements of Li_3_N (fig. S8), we selected hundreds of micrometer-sized Li_3_N particles as coarse particles and micrometer-sized Li_3_N particles as fine particles to construct the graded particle layer. As a result, interface failure is reduced while providing space for dendrite penetration (Fig. 1C and fig. S3B), and the interface maintains intimate interfacial contact after cycling (fig. S9). As presented in Fig. 2 (C to E), the performance of symmetric Li metal batteries is considerably improved, demonstrating stable operation for at least 200 hours at current densities of 0.2 and 0.5 mA cm^−2^ at 27°C, with nearly constant overpotentials maintained. Impressively, the symmetric Li metal battery, featuring this intentional design structure, can operate at an exceptionally high current density of 20 mA cm^−2^ (see Fig. 2E). In a similar way, this design concept can be effectively applied to enhance the electrochemical performance of symmetric Li metal batteries using LGPS SSE (Fig. 2, F to J, and figs. S10 and S11). Drawing from the remarkably improved performance of symmetric Li metal batteries depicted in Fig. 2, it can be inferred that the distribution of both coarse and fine particles within Li_3_N layers can effectively enhance stability at the Li|SSE interface.

**Fig. 3.**
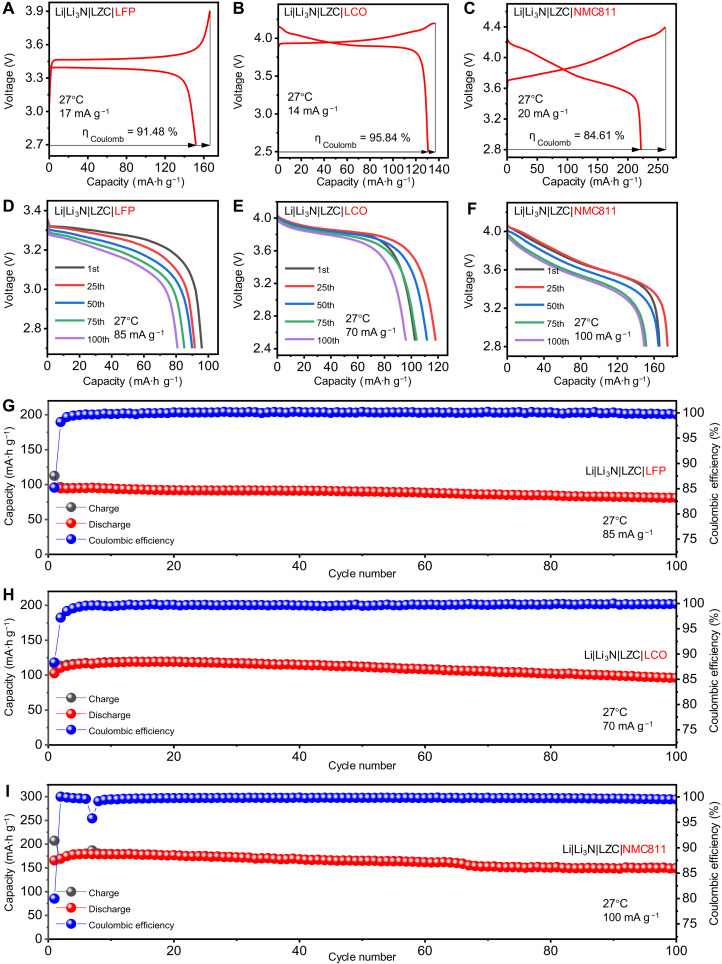
Electrochemical performance of the Li|Li_3_N|LZC|LFP (as well as LCO and NMC811) batteries at 27°C. (**A** to **C**) The initial charge/discharge curves at 17, 14, and 20 mA g^−1^, respectively, with the coulombic efficiency η_Coulomb_ denoted. (**D** to **F**) Discharge curves at 1st, 25th, 50th, 75th, and 100th cycle. (**G** to **I**) Long-term cycling performance at 85, 70, and 100 mA g^−1^, respectively. The Li_3_N layers are composed by fine and coarse Li_3_N particles.

Furthermore, with the intentional design structure of bilayer SSE, ASSBs equipped with various cathodes [LiFePO_4_ (LFP) or LiCoO_2_ (LCO) and LiNi_0.8_Mn_0.1_Co_0.1_O_2_ (NMC811)] and a Li metal anode exhibit stable charge and discharge performance at 27°C, as presented in Fig. 3. When cycled at 17 mA g^−1^ in the voltage range of 2.7 to 3.6 V (versus Li/Li^+^), the Li||LFP battery exhibits an initial coulombic efficiency of 91.48% and delivers a discharge capacity of 152.06 mA·hour g^−1^, as depicted in Fig. 3A. Impressively, the Li||LFP battery maintains a high coulombic efficiency of 99.71% and exhibits a capacity retention of 84.22% after 100 cycles at 85 mA g^−1^, as shown in Fig. 3 (D and G). In addition, the stable cycling performances are observed in the Li||LCO battery. When cycled at 14 mA g^−1^ in the voltage range of 2.5 to 4.2 V (versus Li/Li^+^), the Li||LCO battery exhibits an initial coulombic efficiency of 94.58% and delivers a discharge capacity of 130.54 mA·hour g^−1^, as depicted in Fig. 3B. The long-term cycling performance (Fig. 3, E and H) reveals that the Li||LCO battery maintains a remarkable coulombic efficiency of 99.88% and retains 93.44% of its initial capacity after 100 cycles at 70 mA g^−1^. In addition, the Li||LCO battery with a high cathode mass loading of 10.2 mg cm^−2^ demonstrates stable cycling at 140 mA g^−1^, maintaining a specific capacity of 85.79 mA·hour g^−1^ after 100 cycles, as shown in fig. S12. Furthermore, we paired the Li metal anode with the NMC811 cathode, featuring a higher charging voltage, to construct the Li|Li_3_N|LZC|NMC811 battery that is cycled within the voltage range of 2.8 to 4.4 V (versus Li/Li^+^). Figure 3C illustrates the charge and discharge profiles of the Li||NMC811 battery during its initial cycle at 20 mA g^−1^. In this regime, the Li||NMC811 battery achieves an initial reversible specific capacity of 221.79 mA·hour g^−1^ while maintaining a coulombic efficiency of 84.61%. Besides, Fig. 3 (F and I) showcases the long-term cycling performance of the Li||NMC811 battery, highlighting a coulombic efficiency of 99.51% and a capacity retention of 90.00% after 100 cycles at 100 mA g^−1^. Evidently, this intentional design can be integrated into various ASSB systems such as Li||LFP, Li||LCO, and Li||NMC811, as it enables the realization of dynamic stability for the SSE|Li interface through the graded Li_3_N particle layer.

### Localized distribution of Li dendrites

**Fig. 4.**
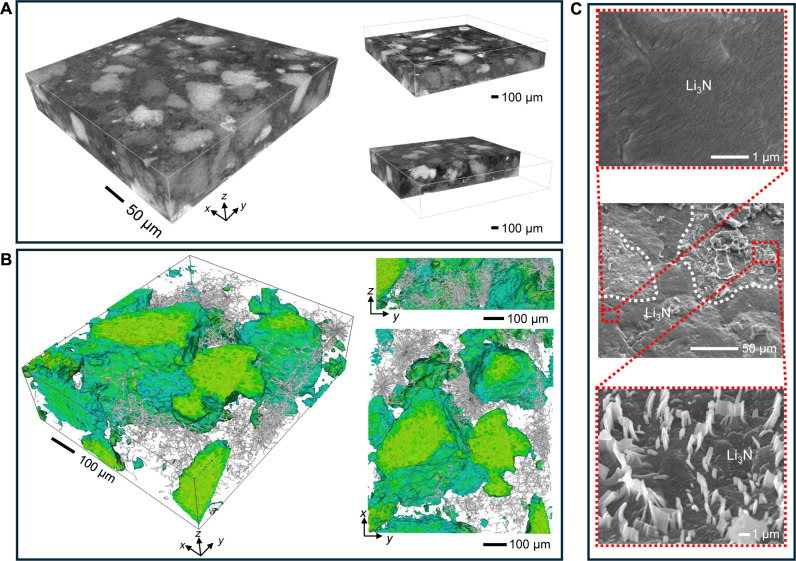
The structure of designed graded Li_3_N particle layer and distribution of Li dendrites. (**A**) XCT images of graded Li_3_N particle layer. (**B**) XCT images of distribution of Li dendrites in graded Li_3_N particle layer, green for coarse Li_3_N particles and silver for trajectories of Li dendrites. (**C**) SEM images of Li dendrites within graded Li_3_N particle layer.

To gain a deeper insight into the influence of the Li_3_N layer structure on the interface stability and the growth behavior of Li dendrites, we used x-ray transmission computed tomography (XCT) to nondestructively observe the three-dimensional configuration of Li_3_N layer. The distribution of the particle size spans from micrometers to hundreds of micrometers (fig. S13). The high-density regions (visible as bright areas in Fig. 4A) primarily consist of coarse particles hundreds of micrometers in size, functioning as mechanical supports. Meanwhile, the low-density regions (visible as dark areas in Fig. 4A) mainly comprise fine particles in the micrometer-sized range, which fill the spaces between the coarse particles, preserving the integrity of the Li_3_N layer and maintaining continuous Li^+^ transport within the bulk and at the interface. Although this intentionally designed layer is susceptible to penetration by Li dendrites (see fig. S14), it demonstrates an effective influence on guiding the growth of Li dendrites in a controllable manner. As a representative case, we disassembled the Li|Li_3_N|LZC|Li_3_N|Li battery after long-term cycling to analyze the underlying mechanisms occurring at the interface. The localized distribution of Li dendrites within the graded Li_3_N particle layer can be distinctly observed in the XCT and scanning electron microscopy (SEM) images. As shown in Fig. 4B, the growth trajectory of Li dendrites appears to circumvent the regions composed of coarse particles, suggesting that the hundreds of micrometer-sized Li_3_N particles effectively impede the intrusion of Li dendrites and confine their distribution into a limited space. The SEM images also verify the localized distribution of Li dendrites. As presented in Fig. 4C, a substantial number of Li dendrites penetrate the regions delineated by white dashed lines, which consist of the fine Li_3_N particles. In contrast, apart from the regions demarcated by dashed lines (i.e., the coarse particles filled areas), there is no penetration of Li dendrites into the Li_3_N layer. Overall, building upon the localized distribution of Li dendrites, it can be concluded that this designed structure of graded Li_3_N particle layer exhibits a dual effect. On one hand, the coarse particles, hundreds of micrometers, offer sufficient rigidity to hinder the growth of Li dendrites. On the other hand, the fine particles, in micrometers, occupying the spaces between coarse particles ensure interface contact and facilitate the controlled propagation of Li dendrites.

**Fig. 5.**
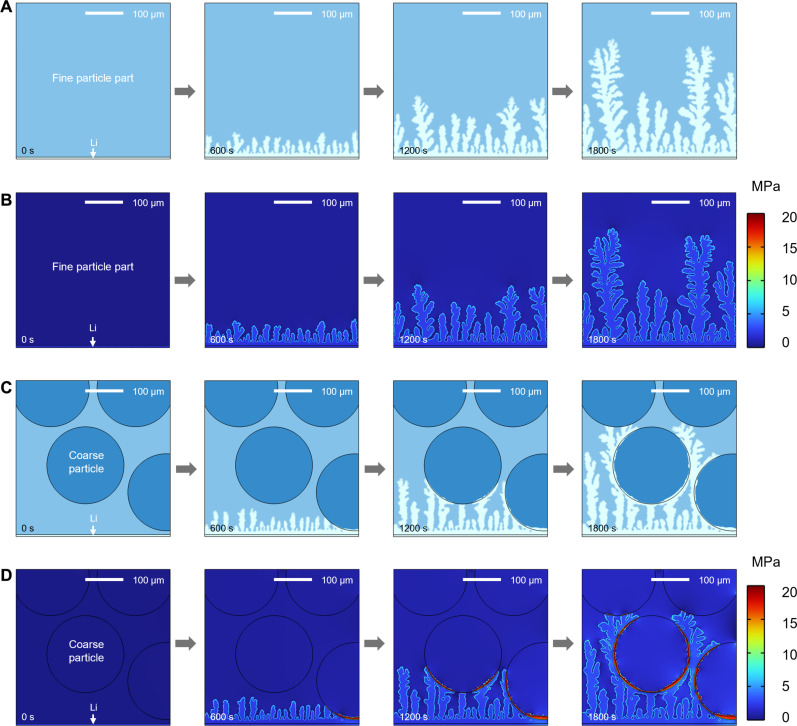
Phase-field simulations for the growth behavior of Li dendrites. (**A** and **B**) Lithium deposition and the variation of the static hydrostatic pressure in Li dendrites versus simulation time in the fine particle system. (**C** and **D**) Lithium deposition and the variation of the static hydrostatic pressure in Li dendrites versus simulation time in the graded particle system. See also movies S1 to S4. In the simulation, a constant overpotential of −0.1 V was applied, closely matching the experimental measurement (Fig. 2D). Both systems maintain consistency in basic properties such as electrical conductivity and Young’s modulus, with the sole distinction being that the nonelastic expansion effects induced by lithium in the first system are caused by fine particles and those in the second system are caused by coarse particles and fine particles.

To further understand the influence of particle distribution in the Li_3_N layer on the behavior of Li dendrites, we used a time-dependent electrochemomechanical phase-field model to simulate the growth of Li dendrites (Fig. 5). In the case of traditional fine particle system, Li dendrites initially appear in needle-like feature. As time progresses, Li deposition gradually forms a substantial branching of dendrites (Fig. 5A), which is in agreement with our observations in SEM images (fig. S7). The uncontrollable growth of Li dendrites can be attributed to the low interfacial stress (~7 MPa) between Li dendrites and fine particles (Fig. 5B), which is insufficient to effectively suppress their propagation (*9*, *25*). In contrast, the behavior of Li dendrites in a graded Li_3_N particle layer, composed of both coarse and fine particles, differs markedly from that observed in the fine particle system (Fig. 5C). The growth state of Li dendrites changes upon contact with coarse particles (at 1200 and 1800 s), displaying a branching structure that selectively bypasses the coarse particles, consistent with XCT and SEM observations (Fig. 4, B and C). The considerable pressure arising within the graded particle structure is the underlying cause of this phenomenon. As Li dendrites propagate, the compressive stress at the interface with coarse particles sharply increases, reaching up to 20 MPa (Fig. 5D). This elevated pressure alters the deposition kinetics of Li dendrites, preventing their penetration into the coarse particle region.

### Self-limiting behavior of Li dendrites

**Fig. 6.**
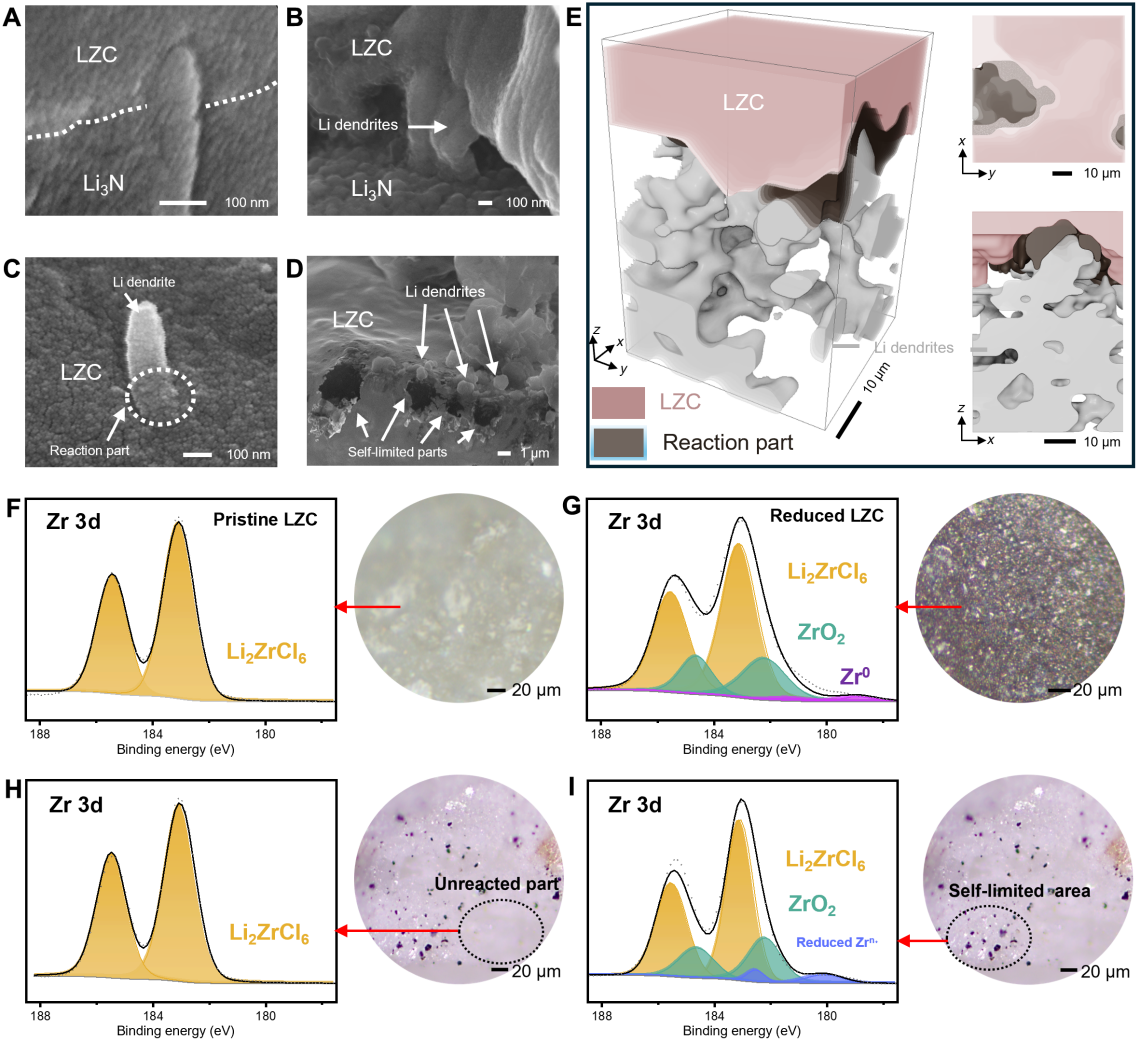
SEM, XCT, and XPS characterization of self-limiting behavior of Li dendrites. (**A** to **D**) SEM images of Li dendrite growth at the interface between Li_3_N and LZC. (**E**) XCT images of self-limiting behavior at the interface between Li_3_N and LZC, pink for LZC, silver for Li dendrites that grow and penetrate graded Li_3_N particle layer, and brown for by-product from the reaction between Li dendrites and LZC. (**F**) XPS of Zr 3d in pristine LZC and (**G**) deteriorated LZC surface area after cycling of Li|LZC|Li cell. (**H**) XPS of Zr 3d in LZC surface area that protected by coarse particles (hundreds of micrometers) in graded Li_3_N particle layer and (**I**) moderately reduced LZC surface area that protected by fine particles (in micrometers) in graded Li_3_N particle layer after cycling of Li|Li_3_N|LZC|Li_3_N|Li. The circular insets in (F) to (I) originate from optical microscope images.

After guiding the growth of Li dendrites through the graded Li_3_N particle layer, their further growth is subsequently limited by the stress induced from their interaction with the less Li-stable LZC electrolyte. As shown in Fig. 6 (A and B) and fig. S15, Li dendrites penetrating the Li_3_N layer become self-limiting within the LZC layer, ceasing their propagation at the transition region between Li_3_N and LZC layers. In addition, Li dendrites are confined in the surface region of LZC (i.e., the decomposition front; Fig. 6C), and the self-limited regions display noticeable dark by-products (Fig. 6D). These findings align with the “expansion screw effect” reported by Ye and Li (*22*), suggesting that local chemical reaction may generate local strain and prevent the further growth of Li dendrites. In addition, the XCT images (Fig. 6E) reveal a transition region (depicted in brown) with a distinct density, located between the Li dendrites (silver) and the LZC layer (pink). This transition region can be categorized as a by-product from the reaction between Li dendrites and LZC, aligning with the findings showcased in the SEM images (Fig. 6, C and D). It should be noted that we intentionally omitted the Li_3_N layer in Fig. 6E using the segmentation function of Avizo software package, to enable clear visualization of the Li dendrites and the interface behavior.

Furthermore, x-ray photoelectron spectroscopy (XPS) analysis on the interface was performed to reveal potential reaction mechanism between Li metal and LZC (Fig. 6, F to I, and fig. S16). In the case of Li|LZC|Li cell after cycling, the XPS data reveal the presence of ZrO_2_ (182.2 eV) and Zr^0^ (178.9 eV) peaks (Fig. 6G), which contrasts with the XPS data for Zr 3d peak (183.1 eV) observed in the pristine LZC (Fig. 6F) (*26*, *27*). The formation of Zr^0^ (i.e., Zr metal) indicates that LZC is reduced by Li metal, and the intrinsic electronic conductivity properties of Zr metal can contribute to the continuous reaction between LZC and Li metal, resulting in the rapid deterioration of the Li|LZC|Li cell (Fig. 2A). It is necessary to clarify that, since there is no O element in the cell, the appearance of the ZrO_2_ signal is probably caused by a reaction of the Zr metal with residual oxygen from either the glovebox or the XPS chamber. This phenomenon is similar to the presence of In_2_O_3_ observed in the Li|Li_3_InCl_6_ (LIC) interface during the in situ XPS measurements (*28*). In contrast, for the Li|Li_3_N|LZC|Li_3_N|Li cell, the XPS signal of Zr 3d (Fig. 6H) obtained from the LZC surface (circled region in the inset) closely resembles the XPS signal of pristine LZC. This observation suggests that the coarse Li_3_N particle regions provide sufficient rigidity to prevent the growth of Li dendrites, thereby avoiding the undesirable reaction between Li dendrites and LZC. The XPS signal of Zr 3d (Fig. 6I) obtained from the dark regions on LZC surface (i.e., shielded by fine Li_3_N particles) reveals the presence of moderately reduced Zr^n+^ (*n* < 4) (180.01 eV) rather than the highly reduced Zr^0^ (178.9 eV). The distinction in XPS signals between Fig. 6G and Fig. 6I implies different interface reaction mechanisms. In the absence of graded Li_3_N particle layer protection, LZC comes into direct contact with Li metal, triggering a chemical reaction as described in Eq. 1. By comparison, for the designed Li_3_N layer, when Li dendrites traverse the fine particle regions, their growth could be restricted by the kinetic stability at the decomposition front, analogous to the expansion screw effect (*22*). Within the self-limited regions of Li dendrite growth, the reduction of Zr^4+^ is suppressed because of local mechanical constraint and the presence of strain (*22*, *29*), corresponding to a possible reaction as described in Eq. 2

$${\text{Li}}_{2}{\text{ZrCl}}_{6}+\text{4Li}\to \text{6LiCl}+{\text{Zr}}^{0}$$ (1)

$${\text{Li}}_{2}{\text{ZrCl}}_{6}+x\text{Li}\to \left(x+2\right)\text{LiCl}+{\text{ZrCl}}_{4-x}\left(0<x<4\right)$$ (2)

**Fig. 7.**
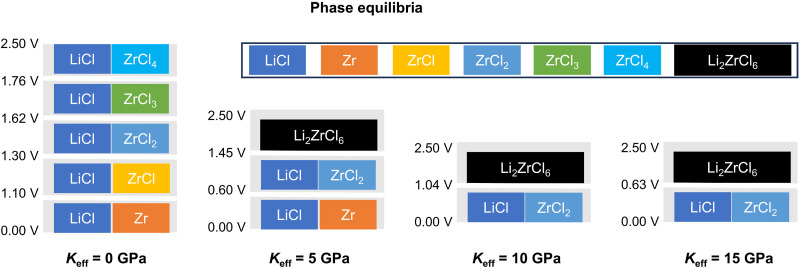
LZC reduction reaction pathways corresponding to different *K*_eff_ and the products in different phase equilibria within each voltage range (voltage versus Li/Li^+^). All decomposition products here are the ground-state phases within each voltage range. The exact decomposition reactions are listed in table S3.

Mechanical stress plays a crucial role in influencing the chemical reaction behaviors at the interface between Li dendrites and SSEs (*29*, *30*). To further investigate interface reaction mechanisms in self-limited regions of Li dendrite growth, we performed density functional theory (DFT) simulation (Fig. 7). The LZC decomposition reaction is simulated by varying the effective bulk modulus *K*_eff_, which quantifies the mechanical constriction and is positively correlated with pressure (*30*). As shown in Fig. 7, as *K*_eff_ increases from 0 to 15 GPa, the decomposition products transition from LiCl and metallic Zr to LiCl and ZrCl_2_, aligning with the experimental XPS findings. This indicates that the pressure in the self-limited regions modifies the chemical reaction behavior at the interface between Li dendrites and SSEs.

**Fig. 8.**
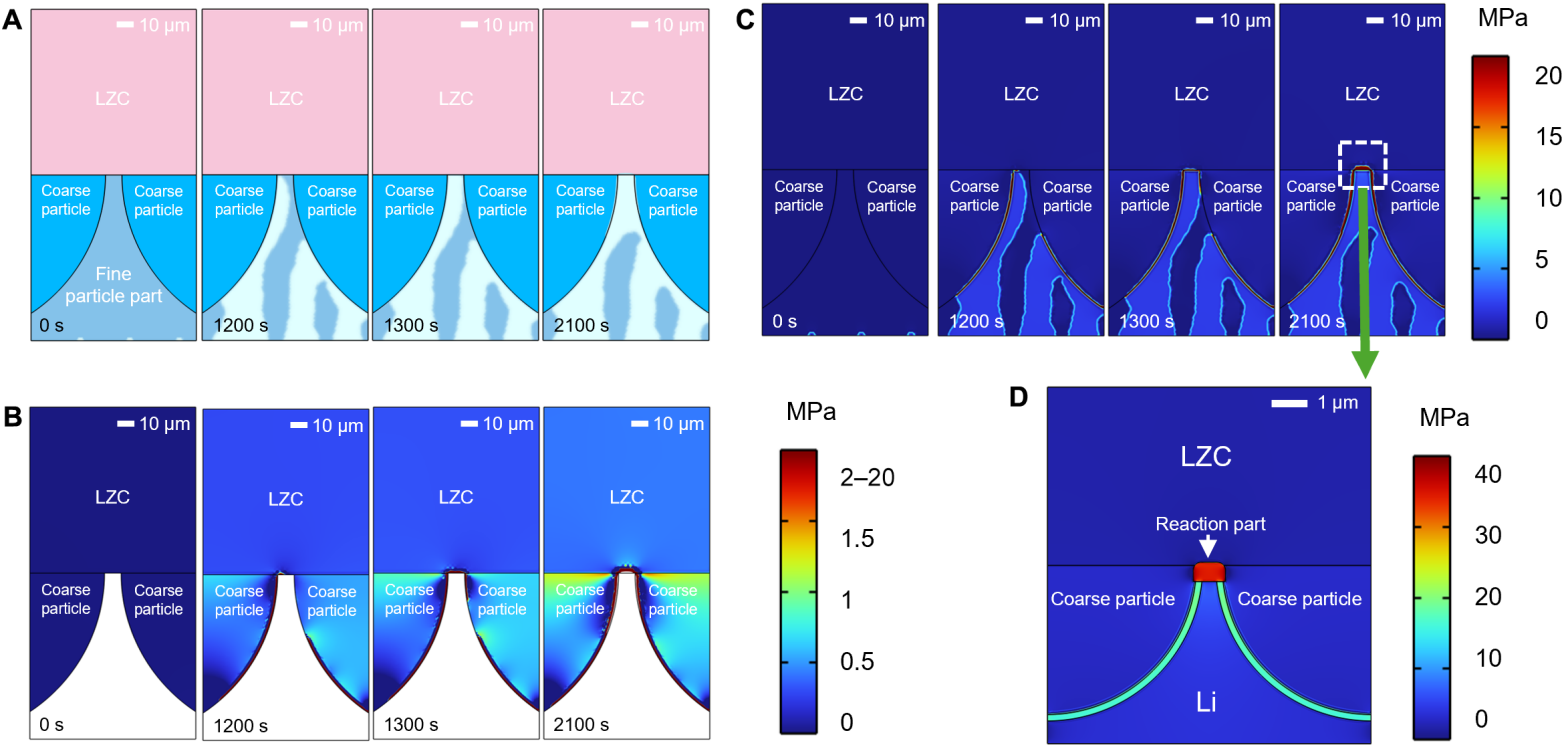
Phase-field simulations for the self-limiting behavior of Li dendrites. (**A**) Lithium deposition at the interface between LZC and Li_3_N. (**B**) The variation of hydrostatic pressure at the interface between LZC and coarse Li_3_N particles. (**C**) The variation of hydrostatic pressure at the interface between LZC and Li_3_N. See also movies S5 to S7. (**D**) Static hydrostatic pressure at the interface between LZC and Li_3_N considering reaction between Li dendrites and LZC layer.

Furthermore, we developed stepwise multiphase models to investigate the self-limiting behavior of Li dendrites, with the first step shown in Fig. 8 (A to C) and the second step in Fig. 8D. During the first step of simulations, the growth of Li dendrites continuously progresses (Fig. 8A). Upon encountering coarse particle regions, Li dendrites are guided to spread along their surfaces and begin to interact with LZC layer at 1200 s. Then, the deposited Li fully covers the interface between the fine Li_3_N particles and LZC (1300 s) and continues to accumulate (2100 s). During the Li deposition process at the interface between LZC and Li_3_N, multiple stress mechanisms can be observed. As shown in Fig. 8B, when Li dendrites grow to the interface with coarse Li_3_N particles, pressure is initially generated at the coarse particle interface. The pressure is then transmitted through the coarse particles to the LZC|Li_3_N interface, resulting in an interfacial pressure exceeding 1 MPa by 2100 s. Notably, to intuitively visualize this pressure transmission mechanism, we intentionally omitted the fine Li_3_N particle region and Li dendrites in Fig. 8B, retaining only LZC and coarse Li_3_N particles. As shown in Fig. 8C, in addition to the pressure induced by the transmission mechanism, continuous deposition of Li dendrites at the interface tip between LZC and the fine particle region (dashed square frame in Fig. 8C) generates stress. The interfacial pressure averages around 10 MPa at 1200 s, increases to ~18 MPa at 1300 s, and further accumulates to about 20 MPa by 2100 s.

In addition, the generation of decomposition products will further increase the pressure at the interface. On the basis of the phase-field simulation from the first step and the decomposition product analysis from XPS and DFT calculations, we conducted the second step of the multiphase simulation, developing a static model (fig. S17). By solving the stress equilibrium equations (Eq. 7), we calculated the static stress to represent the pressure distribution at the interface after the generation of decomposition products (Fig. 8D). The volume expansion effect of the LZC decomposition reaction generates high pressure, which, combined with the pressure distribution from the first-step simulation, results in a pressure of up to 40 MPa at the tip of the interface between LZC and the fine particle part. The elevated pressure further influences the reaction products at the interface between LZC and Li_3_N (Fig. 7) and suppresses Li deposition (*9*, *25*, *31*), resulting in a dynamically stable interface. This explains the self-limiting behavior of Li dendrites observed in experiments, where the growth of Li dendrites is inhibited at the LZC layer (Fig. 6, C and D).

Overall, the combined experimental and computational results provide valuable insights into the self-limiting behavior of Li dendrites at the interface between LZC and Li_3_N, which is governed by the interplay of three types of pressures: (i) the interface-transmitted pressure on the LZC layer from coarse particles induced by Li dendrite growth, (ii) the pressure induced by Li deposition, and (iii) the stress generated by the volume expansion of reaction products. Collectively, these forces effectively suppress Li dendrites and the reduction of LZC, promoting dynamic interfacial stability and facilitating the self-limiting growth of Li dendrites.

It is worth noting that this structure design is not limited to any specific SSEs. The Li_3_N|LZC or Li_3_N|LGPS electrolyte serves as a representative example in this study, demonstrating the advantages of this SSE design principle in regulating Li dendrites. Similarly, we have also validated the applicability of this structural concept to Li_6_PS_5_Cl (LPSCl), where the graded LPSCl particle layer guides the growth of Li dendrites and induces self-limiting behavior at the LPSCl|LZC interface, resulting in a dynamically stable interface (figs. S18 to S20). The concept of using graded particle sizes in the SSE layer to guide Li dendrite growth, coupled with a less Li-stable SSE to limit their growth, is broadly applicable to various types of SSEs—such as halides, oxides, oxyhalides, and sulfides—provided that they can be designed into a feasible structure.

## DISCUSSION

We engineered a structured SSE with graded Li_3_N particle layer, strategically paired with a less Li-stable electrolyte (LZC or LGPS). This design effectively guides Li dendrite growth, localizing their distribution, and achieves dynamic stability and self-limiting behavior. It obviously enhances the performance of symmetric Li metal cells, enabling stable cycling at a high current density of 20 mA cm^−2^ and improving the cycling stability of all-solid-state lithium metal batteries. Our study presents a strategy for regulating Li dendrites and elucidates the mechanisms behind their behavior from guided growth to self-limiting within the designed SSE structure.

## MATERIALS AND METHODS

### Preparation of materials

LZC were synthesized from the stoichiometric amount of LiCl (99%; Aladdin) and ZrCl_4_ (99.9%; Aladdin). The raw materials were mechanochemically milled in the argon-filled atmosphere within a ZrO_2_ jar with ZrO_2_ balls (diameter of 5 mm). The ball-to-powder mass ratio is 20:1 during sample preparation, and the milling was performed in a planetary mill (PULVERISETTE 7 PL, Fritsch) at 500 rpm for 33 hours. The coarse Li_3_N particles in hundreds of micrometers in size (fig. S21A) were synthesized by annealing the Li_3_N (99.9%; Aladdin) at 650°C for 3 hours within a nickel crucible in the argon-filled atmosphere. The fine Li_3_N particles in uniform micrometer size (fig. S21B) were synthesized from Li_3_N (99.9%; Aladdin) by ball milling within a ZrO_2_ jar with ZrO_2_ balls (diameter of 5 mm). The ball-to-powder mass ratio is 20:1 during sample preparation, and the milling was performed at 500 rpm for 16 hours. The Li_3_N layers were fabricated by cold-pressing Li_3_N powder with fine and coarse particles within polyether-ether-ketone (PEEK) molds during the battery assembly process (figs. S22 and S23).

### Material characterization

The morphology of SSEs, Li dendrites, and interface were obtained using an emission SEM (JSM-7610Fplus, Japan). XPS measurements were performed using an x-ray photoelectron spectrometer (PHI 5000 VersaProbe III, Japan). XCT images were acquired using a high-resolution imaging system self-developed by National Center for Applied Mathematics Shenzhen (NCAMS). The self-developed high-resolution imaging system was constructed using the following major components: a panel detector (Shad-o-Box HS, Teledyne DASLA, Canada), a microfocus x-ray source (FOMM 160.02E TT, FineTec, Germany), an x-ray collimator and a motorized rotary stage. The XCT images were imported into Avizo software package (Thermo Fisher Scientific, USA) for three-dimensional visualization, segmentation, and measurements.

### Cell assembly and electrochemical measurements

The symmetric Li metal cells using SSEs (LZC and LGPS; 99.9%, Kejing) were assembled to investigate the stability between SSEs and Li metal. Before assembling the Li|SSE|Li symmetric cells, the electrolyte powders (~100 mg) were pressed into SSE pellets under 4 tons within a PEEK mold with an inner diameter of 10 mm. Then, the Li metal foils (thickness of ~100 μm and diameter of 5 mm) were pressed onto both sides of the SSE pellets. The symmetric cells of Li|Li_3_N|SSE|Li_3_N|Li were assembled to investigate the influence of Li_3_N layer on the SSE|Li interface and the growth of Li dendrites. In a step-by-step process, Li_3_N powder (~60 mg), followed by LZC or LGPS powder (~80 mg), and then Li_3_N powder again (~60 mg) were successively compressed under a pressure of 4 tons within a PEEK mold. Subsequently, Li metal foils (thickness of ~100 μm and diameter of 5 mm) were pressed onto the both sides of sandwich structure electrolyte Li_3_N|SSE|Li_3_N. All the above preparations were performed within a glovebox filled with an argon atmosphere. The galvanostatic cycling at different current densities of the symmetric Li metal cells was conducted on the Wuhan Land battery tester at 27°C.

ASSBs were assembled in PEEK molds using different cathodes, together with Li metal anode and Li_3_N|LZC electrolyte. The cathodes were composed of a mixture containing the active material LFP (Canrd Corp., China), LCO (Canrd Corp., China), or NMC811 (Canrd Corp., China), along with ionic conductor additives (LZC or LIC; MTI Corp., China), and conductive additives [vapor grown carbon fiber (VGCF) or Super P; Canrd Corp., China]. The weight ratios for these mixtures were as follows: For LFP-based cathode, it was LFP:LZC:VGCF = 66:31:3; for LCO-based cathode, it was LCO:LIC:Super P = 70:25:5; and for NMC811-based cathode, it was NMC811:LIC:VGCF = 70:25:5. Before assembling the ASSBs, bilayer electrolyte consisting of Li_3_N (~60 mg) and LZC (or LGPS; ~80 mg) was compacted under a pressure of 3 tons within a PEEK mold with a diameter of 10 mm. Subsequently, the cathode mixture (~1 mg) was pressed under a pressure of 4 tons onto the LZC layer side, and Li metal foil (thickness of ~100 μm and diameter of 10 mm) was affixed to the graded Li_3_N particle layer side by pressing under 0.1 tons to assemble the integrated ASSB. All the above preparations were performed within a glovebox filled with an argon atmosphere. The galvanostatic charge and discharge of ASSBs were conducted on the Wuhan Land battery tester at 27°C.

### Phase-field model for simulating Li dendrite growth

In this electrochemomechanical phase-field modeling, two phase-field order parameters ( ξ and ψ ) are introduced to distinguish the three-phase system: the Li metal ( ξ = 1, ψ = 0), the fine particles ( ξ = 0, ψ = 0), and coarse particles ( ξ = 0, ψ = 1). On the basis of the phase-field model proposed by Chen *et al.* (*32*), the elastic effects are incorporated into the overpotential to elucidate the effect of stress on dendrite morphology in SSEs (*25*). The driving force for the evolution of the phase-field variables is

$$\begin{array}{cc} \frac{\partial \xi }{\partial t} & =-L_{\sigma }(g^{\prime }\left(\xi \right)-\kappa \nabla^{2}\xi +\frac{\partial f_{\text{ns}}\left(\xi \right)}{\partial }\xi )-\\ & L_{\eta }h^{\prime }\left(\xi \right)\left\{\text{exp}\left[\frac{\left(1-\alpha \right)\mathrm{nF}\eta_{a}}{\mathrm{RT}}\right]-c_{+}\text{exp}\left[\frac{-\alpha \mathrm{nF}\eta_{a}-F\eta_{m}}{\mathrm{RT}}\right]\right\} \end{array}$$ (3)

$$\partial \psi_{i}/\partial t=-L_{\sigma }^{s}\frac{\delta F}{\delta \psi }$$ (4)

$L_{\sigma }^{s}$ is the interface mobility, and $L_{\eta }$ is the electrochemical reaction factor. The double-well potential function $g\left(\xi \right)=W\xi^{2}{\left(1-\xi \right)}^{2}$ defines the local energy density, where W is the barrier and κ is the surface energy coefficient. A noise term $f_{\text{ns}}\left(\xi \right)=h^{\prime }\left(\xi \right)A\chi$ is introduced to represent surface heterogeneity, where A and χ are the fluctuation amplitude and the random noise, respectively. An interpolation function $h\left(\xi \right)=\xi^{3}\left(6\xi^{2}-15\xi +10\right)$ is given to ensure that electrochemical reactions occur only at the lithium surface. The $\eta_{a}$ and $\eta_{m}$ are the activation overpotential and the stress-modified overpotential, respectively. The $c_{+}$ is the normalized concentration of Li^+^. The α , *n*, *F*, *R*, and *T* represent the number of symmetry factor, exchanged electrons, the Faraday constant, the gas constant, and the temperature, respectively.

The concentration dynamics are described using the Nernst-Planck equation (*32*)

$$\begin{array}{c} \frac{\partial c_{+}}{\partial t}=\nabla \cdot \left[D^{\text{eff}}\nabla c_{+}+\frac{D^{\text{eff}}c_{+}}{\mathrm{RT}}\mathrm{nF}\nabla \phi \right]-\frac{c_{s}}{c_{0}}\frac{\partial \xi }{\partial t} \end{array}$$ (5)

where $D^{\text{eff}}=h\left(\xi \right)D^{\text{Li}}+\left[1-h\left(\xi \right)\right]D^{\text{SSE}}$ is the effective diffusion coefficient, and $D^{\text{Li}}$ and $D^{\text{SSE}}$ are the diffusion coefficients of the electrode and the electrolyte (*33*), respectively. The $c_{s}$ and $c_{0}$ are the site density of Li metal and the bulk concentration of the electrolyte, respectively.

The electrostatic potential is governed by the Poisson equation, with the source term representing charge annihilation at the reaction interface (*32*), expressed as

$$\nabla \cdot \left\{\sigma^{\text{eff}}\nabla \left[\text{ϕ}\left(r,t\right)\right]\right\}=\mathrm{nF}c_{s}\partial \xi /\partial t$$ (6)

where $\sigma^{\text{eff}}=h\left(\xi \right)\sigma^{\text{Li}}+\left[1-h\left(\xi \right)\right]{\text{σ}}^{\text{SSE}}$ is the effective conductivity and $\sigma^{\text{Li}}$ and $\sigma^{\text{SSE}}$ are the conductivity of Li and electrolyte, respectively.

The stress equilibrium equation is expressed as

∇σ=0 (7)

where $\sigma =C^{\mathrm{eff}}\cdot \varepsilon^{\mathrm{el}}=C^{\mathrm{eff}}\cdot \left(\varepsilon -\varepsilon^{0}\right)$ . The effective elastic modulus $C^{\mathrm{eff}}\left(\xi \right)$ is termed as $C^{\mathrm{eff}}\left(\xi \right)=h\left(\xi \right)C_{\text{ijkl}}^{\text{Li}}+\left[1-h\left(\xi \right)\right]C_{\text{ijkl}}^{\text{SSE}}$ , where $C_{\text{ijkl}}^{\text{Li}}$ and $C_{\text{ijkl}}^{\text{SSE}}$ are the elastic modulus of Li and the electrolyte, respectively. The nonelastic strain can be expressed as $\varepsilon^{0}=K_{\mathrm{ii}}\xi$ , where $K_{\mathrm{ii}}$ is a constant diagonal matrix.

The modified mechanical overpotential is now expressed as

$$\eta_{m}=C\frac{Pv_{m,\text{Li}}}{F}$$ (8)

where $P=1/3\sigma_{\mathrm{ii}}$ represents the hydrostatic stress acting on the front of the electrochemical reaction. $v_{m,\text{Li}}$ is the molar volume of Li metal. *C* is the mechanical overpotential correction factor.

The simulation was conducted using COMSOL Multiphysics 6.1, using finite element analysis. The model involves five governing equations: phase-field order parameters ( ξ , ψ ), Li^+^ concentration ( $c_{+}$ ), electric field ( ϕ ), and solid mechanics equations. The detailed parameters used for the phase-field model are listed in table S1.

### DFT calculations

All calculations were performed using the Vienna Ab Initio Simulation Package (VASP) using the recommended projector augmented wave pseudopotentials. All DFT calculations were performed following the Materials Project calculation parameters (*34*). An energy cutoff of 520 eV with a *k*-point mesh of 1000 *k*-points per atom was used, and the VASP-recommended pseudopotentials were used. Mechanically constrained phase diagrams were calculated using Lagrange minimization schemes for effective moduli of 0, 5, 10, and 15 GPa (*35*).

The initial crystal structure of LZC, space group *C*2/*m*, exhibits disorder from experimental results. Therefore, all symmetrically distinct atomic configurations must first be enumerated, and their energies were calculated to identify the lowest-energy ordering (or at least a reasonably low-energy ordering). The lowest-energy structure was then used as input for subsequent calculations and analyses. The other crystal structures spanning the Li-Zr-Cl phase space were obtained from the Materials Project (*34*).

### Phase-field model for simulating self-limiting behavior of Li dendrites

On the basis of the results of phase-field model of the interface between graded Li_3_N particles and LZC (dashed square frame in Fig. 8C) and DFT (Fig. 7), we define the lithium phase as ξ , where its evolution follows the Allen-Cahn equation (*25*); the fine particle part as $\psi_{1}$ , which is covered by deposited Li; the coarse particles as $\psi_{2}$ ; and the LZC phase as $\psi_{3}$ ,. The detailed characteristics of these phases and interfaces are illustrated in fig. S17. On the basis of the specific reaction conditions, $\psi_{4}$ represents the reaction part, while $\psi_{5}$ denotes the interface between Li and coarse Li_3_N particles. Roller constraint is applied on both the left and right sides. A fixed displacement boundary condition of *u* = 0 is set for both the top (electrolyte side) and bottom (electrode side). Simulation-related parameters are provided in table S2, and other coefficients are the same as in table S1. According to experimental observations (Fig. 6D), it is inferred that the reaction parts exhibit a higher expansion effect; hence, its expansion coefficient is set to two times greater than that of the graded Li_3_N particle phase. The control equations adopted in this model are identical to those of the phase-field model described above.
