# Supplementary material for: Dynamic control of lithium dendrite growth with sequential guiding and limiting in all-solid-state batteries
Source: Sci Adv. 2025 Aug 20;11(34):eadw9590. doi: 10.1126/sciadv.adw9590 (PMC12366673; doi:10.1126/sciadv.adw9590)
Supplement: Supplementary file 1 — Figs. S1 to S23 Tables S1 to S3 Legends for movies S1 to S7 [file sciadv.adw9590_sm.pdf]

Science Advances
AAAS

Supplementary Materials for

Dynamic control of lithium dendrite growth with sequential guiding and limiting in all-solid-state batteries

Longbang Di *et al.*

Corresponding author: Lei Gao, gaolei2018@pku.edu.cn; Yunxing Zuo, zuoyunxing@eacomp.com; Songbai Han, hansb@sustech.edu.cn; Ruqiang Zou, rzou@pku.edu.cn

Sci. Adv. **11**, eadw9590 (2025)
DOI: 10.1126/sciadv.adw9590

The PDF file includes:

Figs. S1 to S23
Tables S1 to S3
Legends for movies S1 to S7

Other Supplementary Material for this manuscript includes the following:

Movies S1 to S7

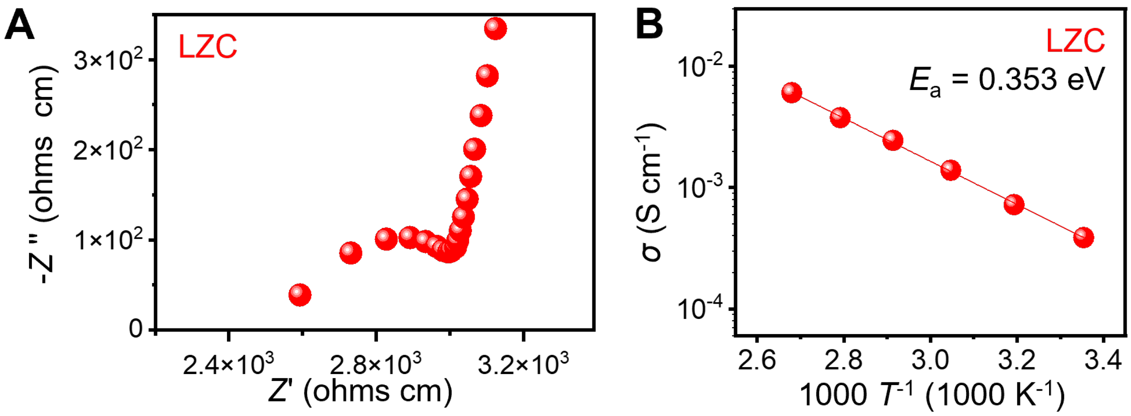

Fig. S1. Ionic conductivity analysis of LZC. (**A**) The Nyquist plot and (**B**) Arrhenius ionic conductivity plot of LZC. The Nyquist plot was processed based on the formula: $Z = (Z_0 \times S) / l$, to eliminate the effect of SSE pellet thickness and area on the impedance. The ionic conductivity of LZC is 3.87×10^{-4} S cm^{-1} at RT.

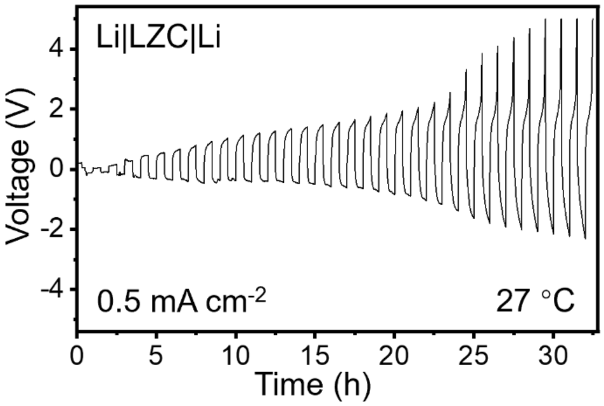

Fig. S2. The performance of Li|LZC|Li cell cycled at 0.5 mA cm^{-2} at 27 °C for 1 h in each cycle.

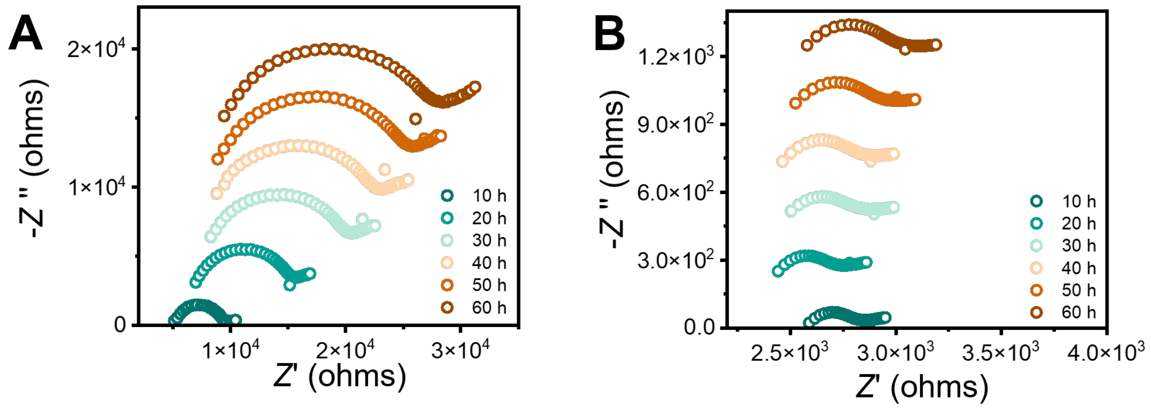

Fig. S3. Electrochemical Impedance Spectroscopy analysis of Li|SSEs|Li cells. (**A**) Symmetric Li metal cell employing LZC as SSE. (**B**) The Li|Li_3N|LZC|Li_3N|Li symmetric cell, in which the Li_3N are composed of fine and coarse Li_3N particles.

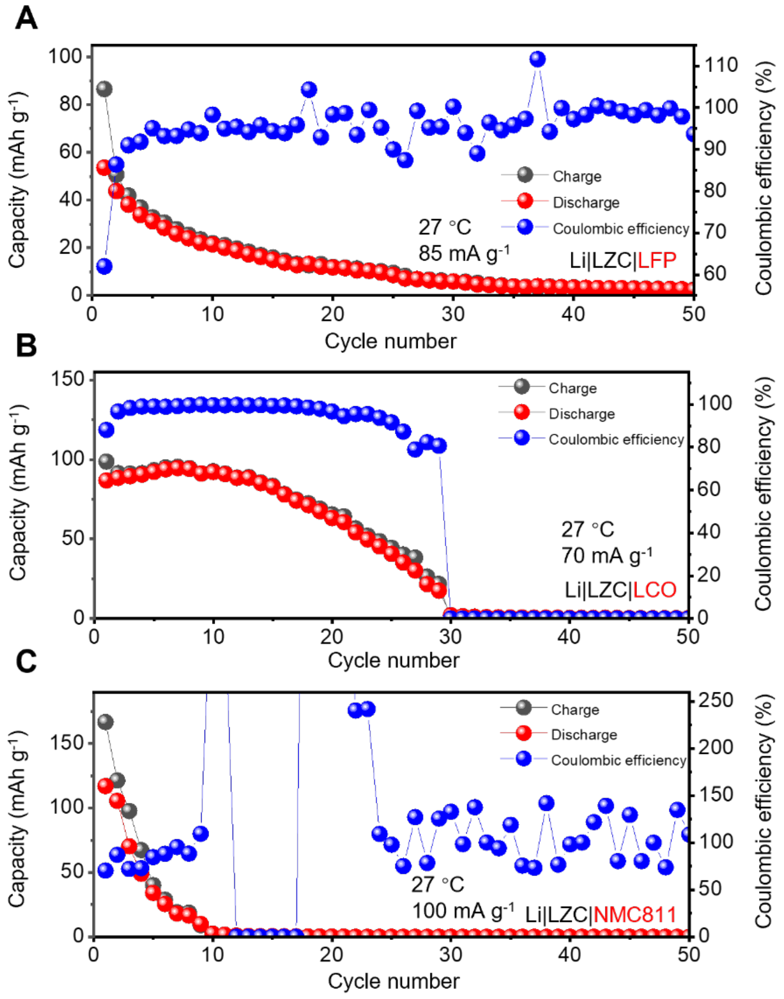

Fig. S4. Electrochemical performance of the Li|LZC|LFP (as well as LCO and NMC811) batteries. (**A**–**C**) Cycling performance at 85, 70 and 100 mA g^{-1}, respectively.

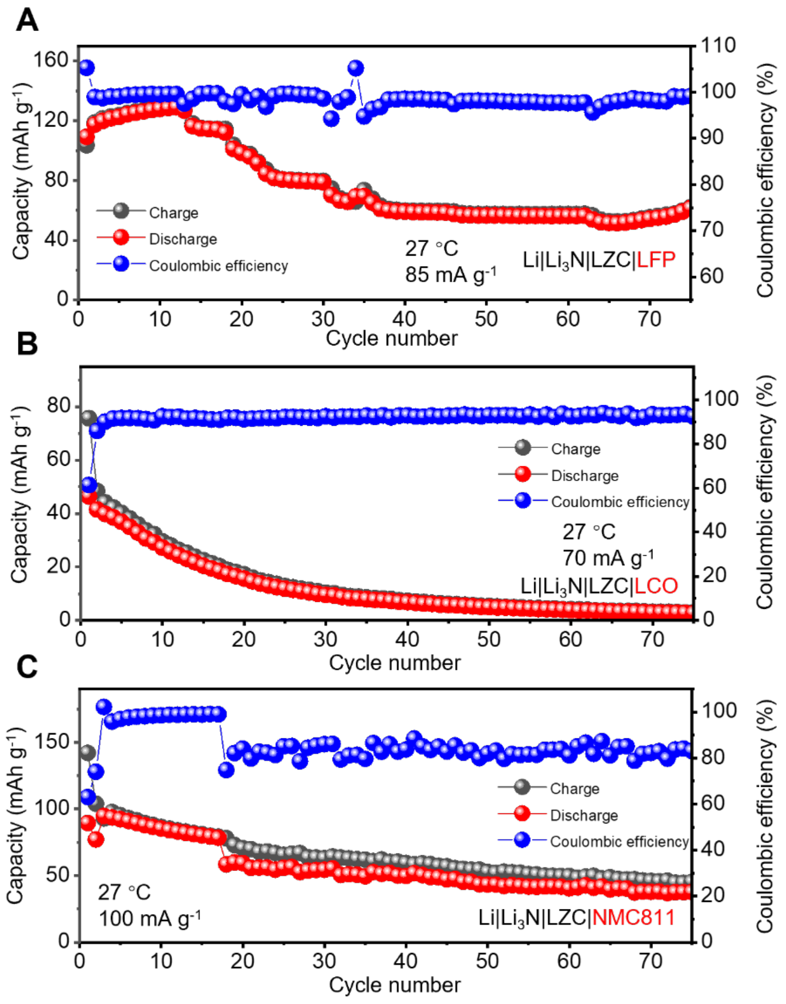

Fig. S5. Electrochemical performance of the Li|Li_3N|LZC|LFP (as well as LCO and NMC811) batteries at 27 °C. (**A–C**) Cycling performance at 85, 70 and 100 mA g^{-1}, respectively, in which the Li_3N layers are composed by fine Li_3N particles.

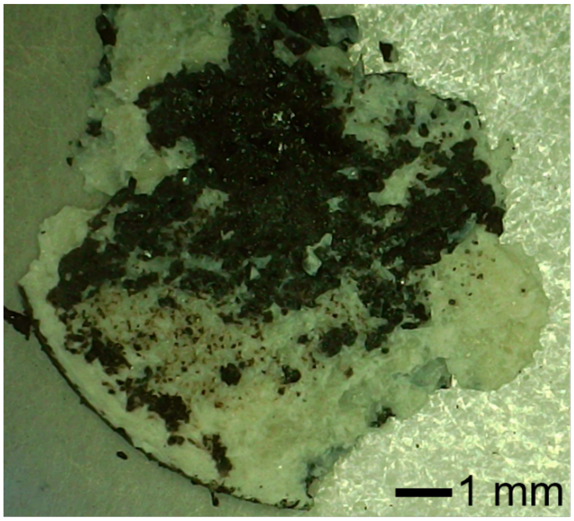

Fig. S6. The optical microscope image of SSE pellet disassembled from Li|Li_3N|LZC|Li_3N|Li cell after cycling, in which the Li_3N layers are composed by fine Li_3N particles.

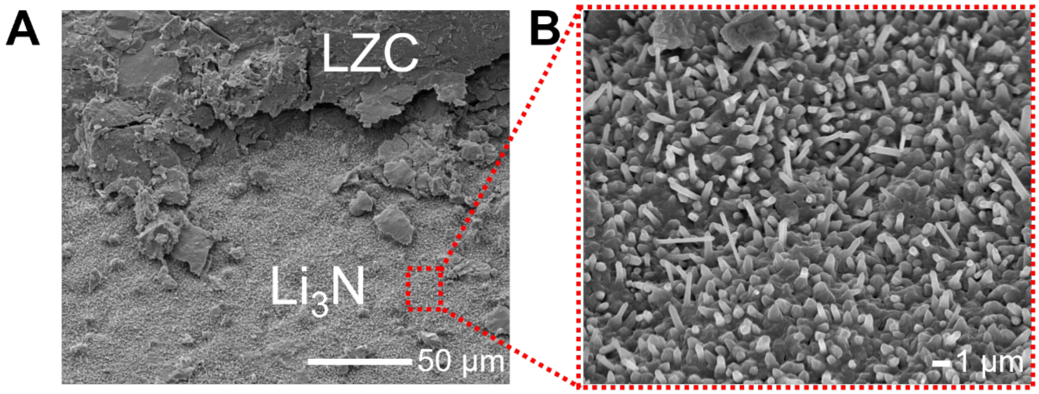

Fig. S7. SEM images of SSE pellets disassembled from Li|Li_3N|LZC|Li_3N|Li cell after cycling. (**A**) The SEM image of interface between LZC and Li_3N. (**B**) Magnified image of Li_3N surface, in which the Li_3N layer is comprised of uniformly sized microns particles.

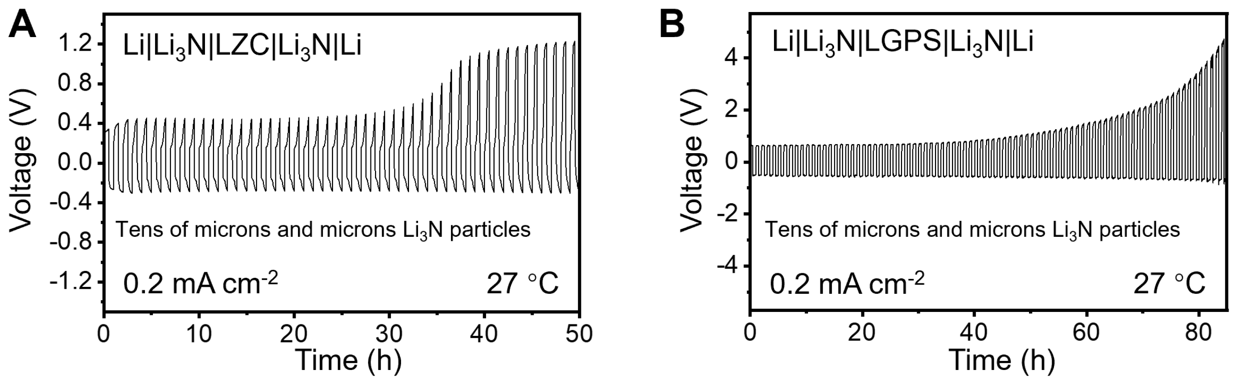

Fig. S8. The performance of Li|SSEs|Li cells, cycling at 0.2 mA cm^{-2} at 27 °C for 1 h in each cycle. (**A**) The Li|Li_3N|LZC|Li_3N|Li symmetric cell. (**B**) The Li|Li_3N|LGPS|Li_3N|Li symmetric cell, in which the Li_3N layers are composed of tens of microns and microns Li_3N particles.

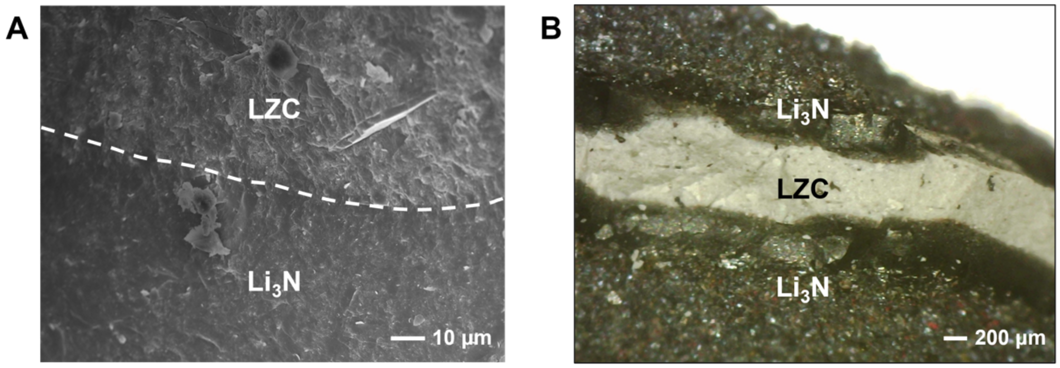

Fig. S9. Images of the LZC|Li_3N interface disassembled from symmetric Li metal cell after cycling. (**A**) SEM image and (**B**) optical microscopy image, in which the Li_3N layers are composed by graded particles in hundreds of microns and microns.

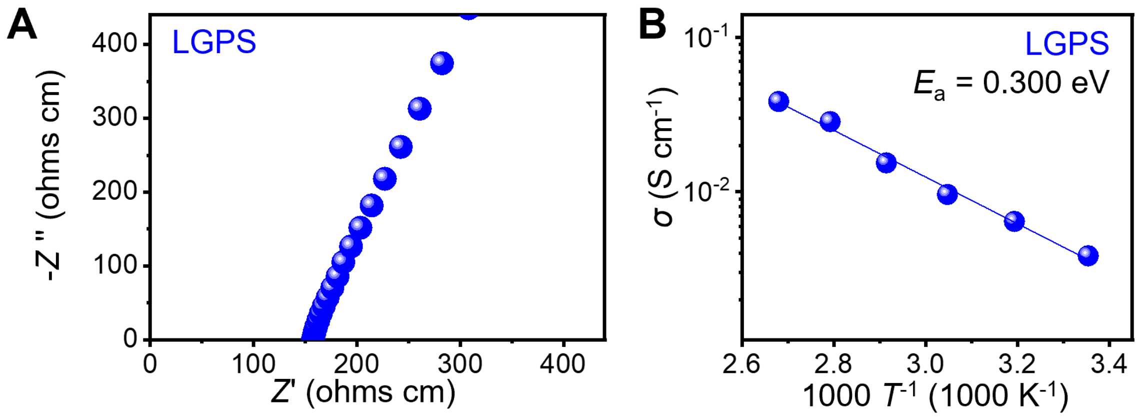

Fig. S10. Ionic conductivity analysis of LGPS. The Nyquist plot (**A**) and Arrhenius ionic conductivity plot (**B**) of LGPS. The Nyquist plot was processed based on the formula: $Z = (Z_0 \times S) / l$, to eliminate the effect of SSE pellet thickness and area on the impedance. The ionic conductivity of LGPS is 6.40×10^{-3} S cm^{-1} at RT.

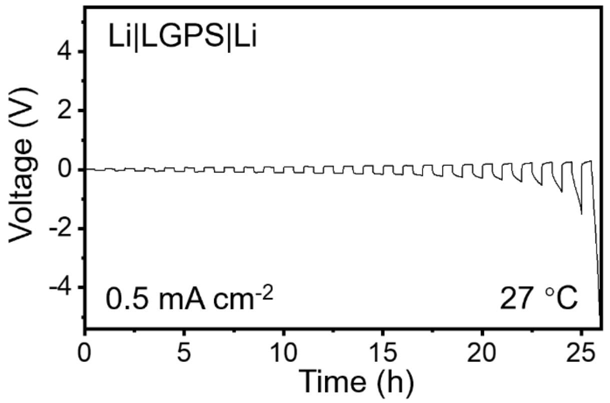

Fig. S11. The performance of Li|LGPS|Li cell cycled at 0.5 mA cm^{-2} at 27 °C for 1 h in each cycle.

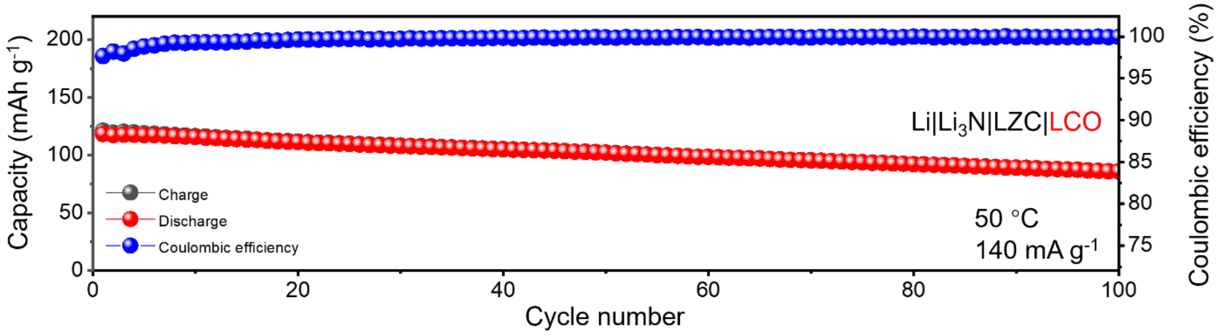

Fig. S12. Long-term cycling performance of Li|Li_3N|LZC|LCO cell at 50 °C and a current density of 140 mA g^{-1} with a high cathode mass loading of 10.2 mg cm^{-2}, in which the Li_3N layers consist of fine and coarse particles.

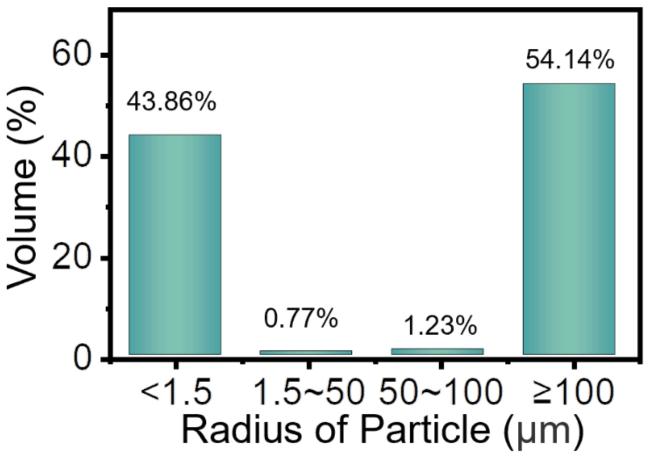

Fig. S13. The proportion of graded Li_3N particles by volume of different radii. The graded Li_3N layer consists predominantly of particles <1.5 μm (43.83%) and >100 μm (54.14%), with only 0.77% (1.5–50 μm) and 1.23% (50–100 μm) arising from the fragmentation of the hundreds of microns sized Li_3N particles during pellet pressing process.

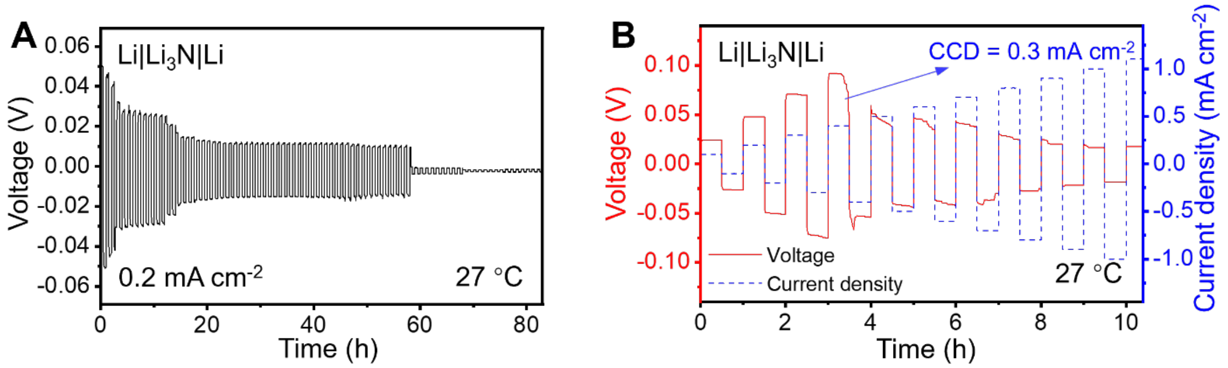

Fig. S14. The performance of symmetric Li metal cells employing graded Li_3N particle layers. (**A**) Symmetric Li metal cell using Li_3N as electrolytes cycled at 0.2 mA cm^{-2} and 27 °C for 1 h in each cycle. (**B**) Critical current density (CCD) of graded Li_3N particle layer. The Li_3N layers are composed by graded particles in hundreds of microns and microns.

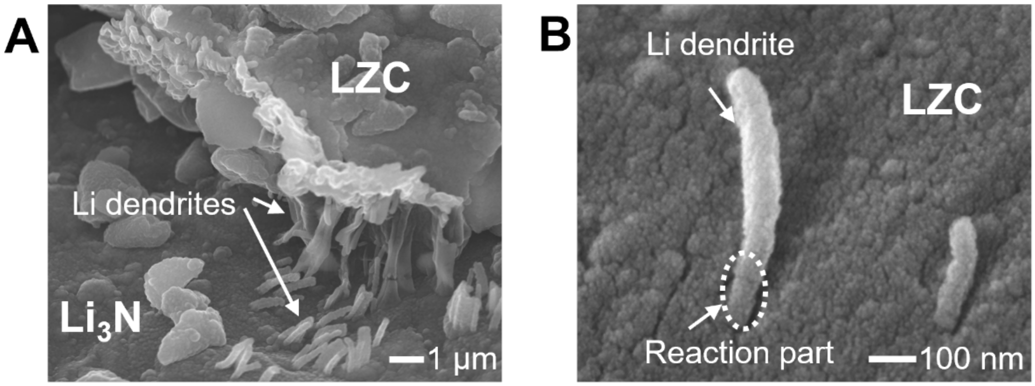

Fig. S15. SEM images of the LZC|Li_3N interface disassembled from symmetric Li metal cell after cycling. The Li_3N layers are composed by graded particles in hundreds of microns and microns.

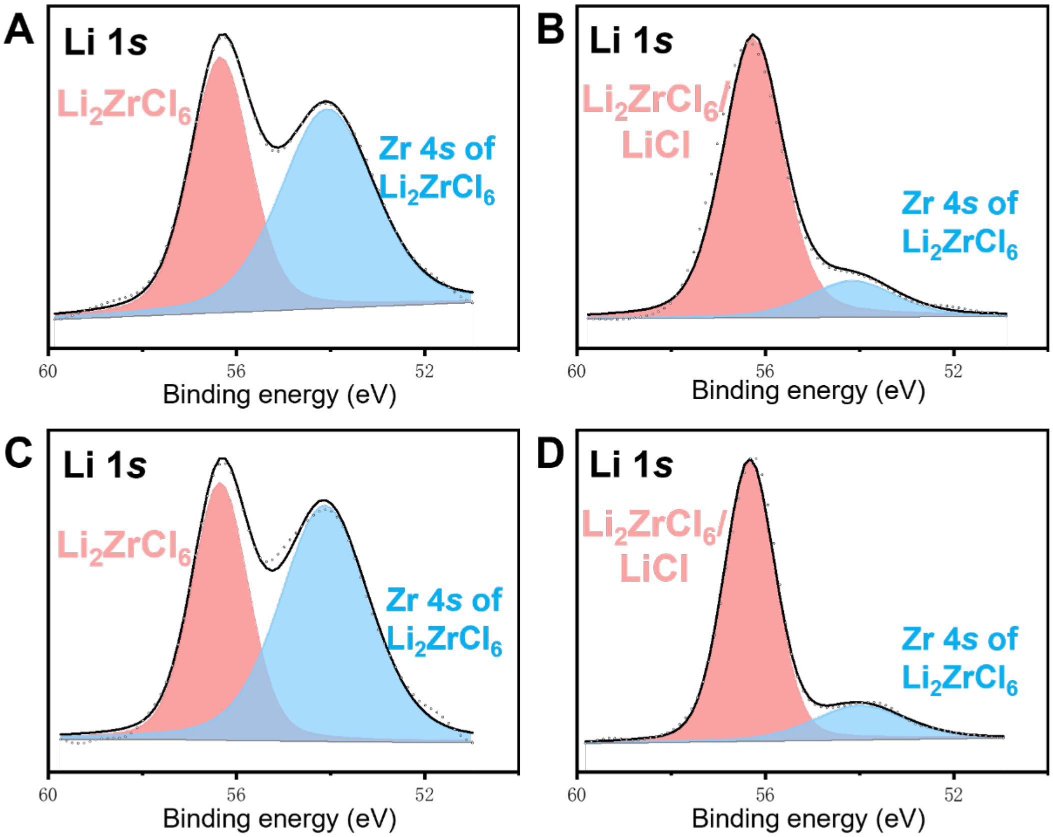

Fig. S16. XPS characterization of LZC interface. (**A**) XPS of Li 1*s* in pristine LZC, and (**B**) deteriorated LZC surface area after cycling of Li|LZC|Li cell. (**C**) XPS of Li 1*s* in LZC surface area that protected by coarse particles (hundreds of microns) in graded Li_3N particle layer, and (**D**) moderately reduced LZC surface area that protected by fine particles (microns) in graded Li_3N particle layer after cycling of Li|Li_3N|LZC|Li_3N|Li. The Li 1*s* peak at 56.4 eV is attributed to Li–Cl in either LZC or LiCl, and an additional peak at 54.1 eV corresponds to the Zr 4*s* signal from Zr^{4+} in the LZC electrolyte.

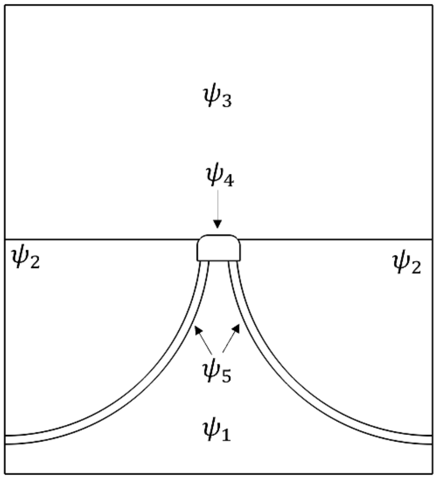

Fig. S17. The model for the phase field simulation at the interface between graded Li_3N particles and LZC. The simulation-related parameters are listed in Table S2.

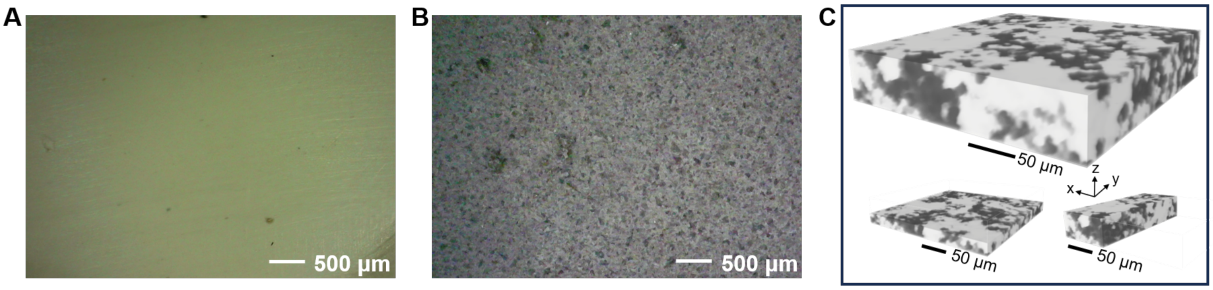

Fig. S18. Images of the LPSCl layer. The optical microscopy images of the layers consist of (**A**) uniform fine particles and (**B**) graded-particles including both fine and coarse particles. (**C**) CT image corresponding to the graded-particle LPSCl layer.

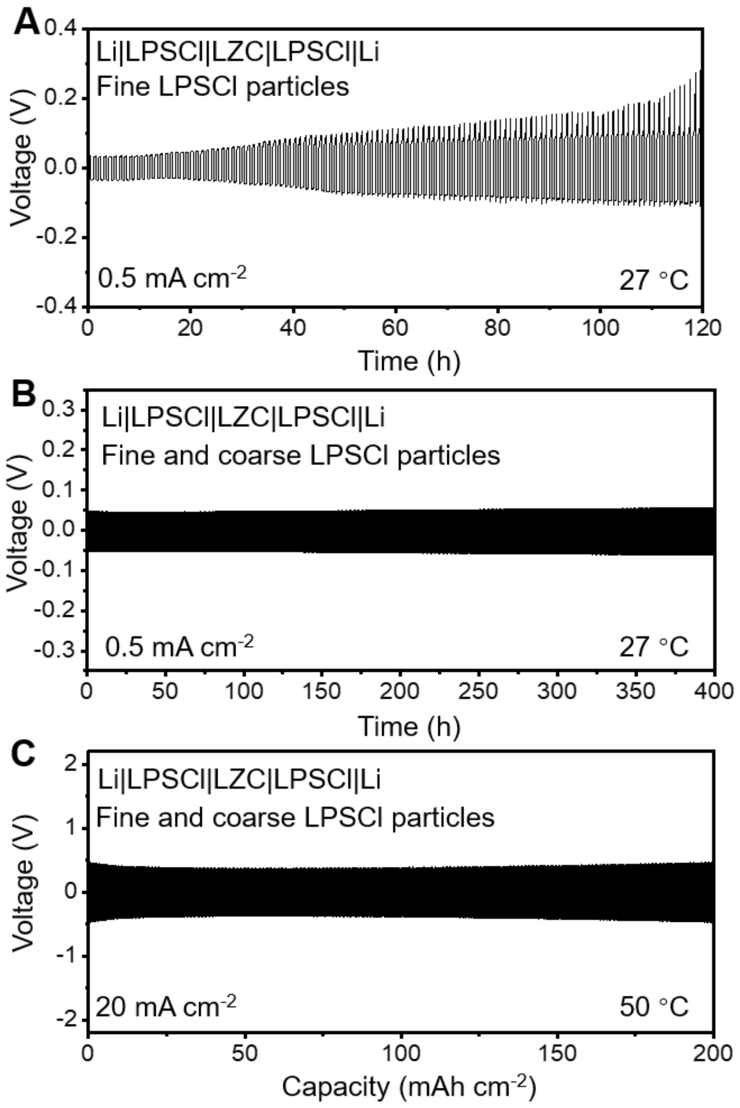

Fig. S19. The performance of Li|LPSCl|LZC|LPSCl|Li symmetric cells. The LPSCl layers in (**A**) are composed of fine LPSCl particles, whereas those in (**B**) and (**C**) consist of both fine and coarse LPSCl particles.

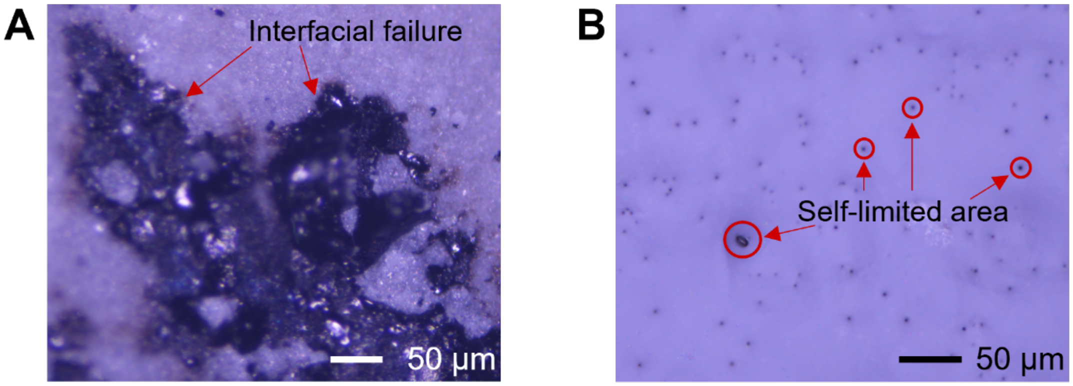

Fig. S20. Optical microscope images of the LZC electrolyte surface after disassembling the cycled Li|LPSCl|LZC|LPSCl|Li symmetric cells. The LPSCl layers in (**A**) are composed of fine LPSCl particles, while those in (**B**) comprise a mixture of fine and coarse LPSCl particles.

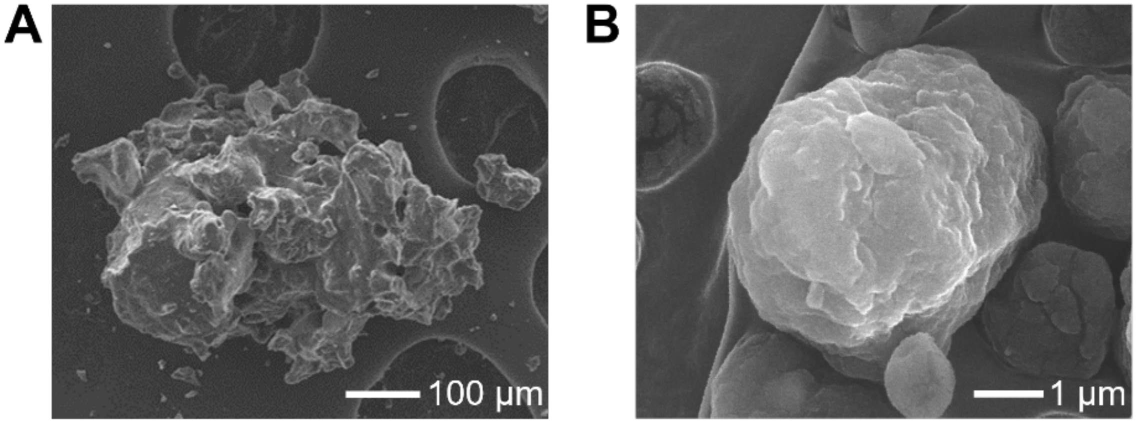

Fig. S21. SEM images of Li_3N particles. (**A**) Hundreds of microns Li_3N particle. (**B**) Microns Li_3N particle.

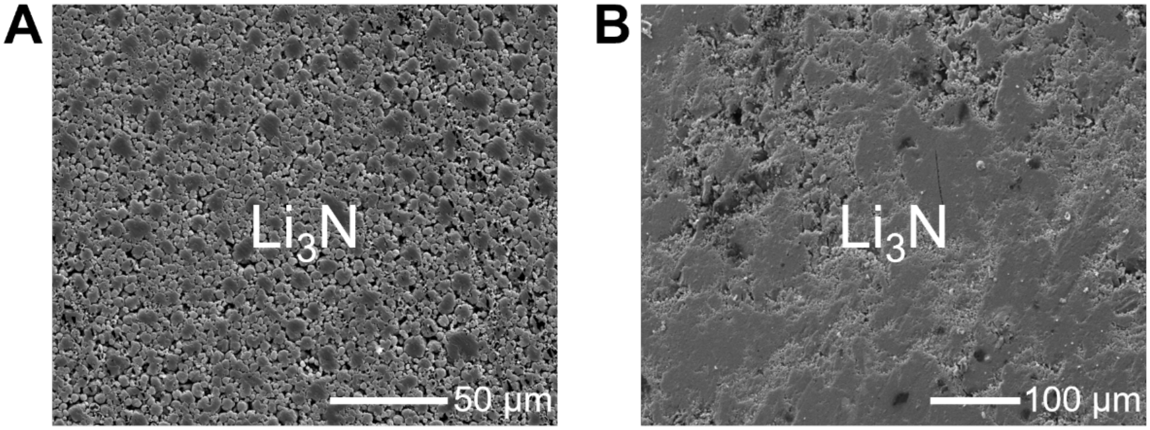

Fig. S22. SEM images of Li_3N pellets. (**A**) Li_3N pellet composed by fine particles in microns. (**B**) Li_3N pellet composed by graded particles in hundreds of microns and microns.

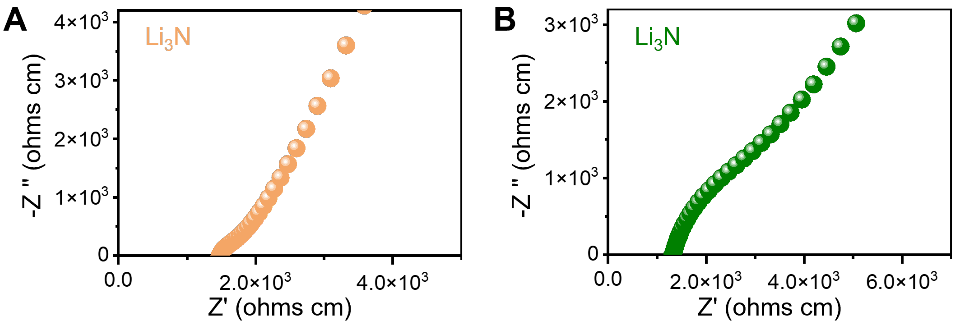

Fig. S23. The Nyquist plots of Li_3N layer. (**A**) The Nyquist plot of Li_3N layer composed by fine particles. (**B**) The Nyquist plots of Li_3N layer composed by fine and coarse particles. The Nyquist plot was processed based on the formula: $Z = (Z_0 \times S) / l$, to eliminate the effect of SSE pellet thickness and area on the impedance. The ionic conductivities of fine Li_3N particle layer and the graded Li_3N particle layer are 5.98×10^{-4} S cm^{-1} and 5.14×10^{-4} S cm^{-1} at RT, respectively.

Table S1. Phase-field simulation parameters

	Symbol	Unit	Real Value		
			Li	Fine particle part	Coarse particle
System Size	l	μm	410*400		
Time step	Δt	s	1		
Barrier Height	W	J/m^3	1.74e6		
Gradient Coefficient	κ_0	J/m	8.7e-7		
Interface energy	γ	J/m^2	0.29		
Interface width	d_0	μm	0.5		
Interface mobility	$L\sigma$	$m^3/(J{\cdot}s)$	4e-8	0	0
Reaction constant	$L\eta$	1/s	0.04	-	-
Anisotropy factor	δ	-	0.05		
symmetry mode	ω	-	4		
Conductivity	σ	S/m	1e7	0.1	0.1
Young's Modulus	E	Pa	5e9	86e9	86e9
Poisson's Ratio	v	-	0.362	0.24	0.24
Symmetric factor	α	-	0.5		
Stress correction term	C	-	10		
Bulk Li-ion concentration	Cs	mol/m^3	76.4e3	21.8e3	21.8e3
Diffusion coefficient	D	m^2/s	1e-15	2.24e-12	2.24e-12
Inelastic strain component	K_{11}	-	5e-5	5e-5	5e-4
	K_{22}	-	5e-5	5e-5	5e-4
	K_{33}	-	5e-4	5e-4	5e-3
Exchange electron numbers	n		1		
Faraday constant	F	C/mol	96500		
Gas constant	R	J/mol/K	8.314		
Temperature	T	K	300.15		
Radius	r	μm			>= 100
Fluctuation amplitude	A	J/m^3	1e5		

Table S2. Simulation-related parameters of the Li-Li_3N-LZC system

	Li	Coarse particle	LZC	Reaction part	Li-Coarse particle
	ξ	ψ_2	ψ_3	ψ_4	ψ_5
Young's modulus (GPa)	5	86	22	52	5
Poisson's ratio	0.362	0.24	0.24	0.24	0.362
K_{11}	5e-5	5e-4	1e-3	1e-3	5e-4
K_{22}	5e-5	5e-4	1e-3	1e-3	5e-4
K_{33}	5e-4	5e-3	1e-2	1e-2	5e-3
σ (S/m)	1e7	1e-1	3.87e-2	3.87e-2	1e7
D (m^2/s)	1e-15	2.24e-12	7.5e-13	7.5e-13	1e-15

Table S3. LZC decomposition products at different K_{eff}

(**A**) K_{eff} = 0 GPa

Voltage *vs*. Li/Li$^+$	LZC + *x* Li	Decomposition products
2.50 V	Li_2ZrCl_6 + 1.78e-14 Li	2 LiCl + $ZrCl_4$
2.06 V	Li_2ZrCl_6 + 1.78e-14 Li	2 LiCl + $ZrCl_4$
2.05 V	Li_2ZrCl_6 + 1.69e-14 Li	2 LiCl + $ZrCl_4$
2.02 V	Li_2ZrCl_6 + 1.69e-14 Li	2 LiCl + $ZrCl_4$
2.01 V	Li_2ZrCl_6 + 1.02e-14 Li	2 LiCl + $ZrCl_4$
1.96 V	Li_2ZrCl_6 + 1.02e-14 Li	2 LiCl + $ZrCl_4$
1.95 V	Li_2ZrCl_6 + 7.11e-15 Li	2 LiCl + $ZrCl_4$
1.78 V	Li_2ZrCl_6 + 7.11e-15 Li	2 LiCl + $ZrCl_4$
1.77 V	Li_2ZrCl_6 + 8.88e-16 Li	2 LiCl + $ZrCl_4$
1.76 V	Li_2ZrCl_6 + 8.88e-16 Li	2 LiCl + $ZrCl_4$
1.75 V	Li_2ZrCl_6 + Li	3 LiCl + $ZrCl_3$
1.63 V	Li_2ZrCl_6 + Li	3 LiCl + $ZrCl_3$
1.62 V	Li_2ZrCl_6 + 2 Li	4 LiCl + $ZrCl_2$
1.30 V	Li_2ZrCl_6 + 2 Li	4 LiCl + $ZrCl_2$
1.29 V	Li_2ZrCl_6 + 3 Li	5 LiCl + ZrCl
1.10 V	Li_2ZrCl_6 + 3 Li	5 LiCl + ZrCl
1.09 V	Li_2ZrCl_6 + 4 Li	6 LiCl + Zr
0.00 V	Li_2ZrCl_6 + 4 Li	6 LiCl + Zr

(**B**) K_{eff} = 5 GPa

Voltage *vs*. Li/Li$^+$	LZC + *x* Li	Decomposition products
2.50 V	Stable	Li_2ZrCl_6
1.45 V	Stable	Li_2ZrCl_6
1.44 V	Li_2ZrCl_6 + 2 Li	4 LiCl + $ZrCl_2$
0.60 V	Li_2ZrCl_6 + 2 Li	4 LiCl + $ZrCl_2$
0.59 V	Li_2ZrCl_6 + 4 Li	6 LiCl + Zr
0.00 V	Li_2ZrCl_6 + 4 Li	6 LiCl + Zr

(**C**) K_{eff} = 10 GPa

Voltage *vs*. Li/Li$^+$	LZC + *x*Li	Decomposition products
2.50 V	Stable	Li_2ZrCl_6
1.04 V	Stable	Li_2ZrCl_6
1.03 V	Li_2ZrCl_6 + 2 Li	4 LiCl + $ZrCl_2$
0.00 V	Li_2ZrCl_6 + 2 Li	4 LiCl + $ZrCl_2$

(**D**) K_{eff} = 15 GPa

Voltage *vs*. Li/Li^+	LZC + *x*Li	Decomposition products
2.50 V	Stable	Li_2ZrCl_6
0.63 V	Stable	Li_2ZrCl_6
0.62 V	Li_2ZrCl_6 + 2 Li	4 LiCl + $ZrCl_2$
0.00 V	Li_2ZrCl_6 + 2 Li	4 LiCl + $ZrCl_2$

Movie S1. Video of phase-field simulations for the growth behavior of Li dendrites.
Lithium deposition versus simulation time in the fine particle system.

Movie S2. Video of phase-field simulations for the growth behavior of Li dendrites.
The variation of the static hydrostatic pressure in Li dendrites versus simulation time in the fine particle system.

Movie S3. Video of phase-field simulations for the growth behavior of Li dendrites.
Lithium deposition versus simulation time in the graded particle system.

Movie S4. Video of phase-field simulations for the growth behavior of Li dendrites.
The variation of the static hydrostatic pressure in Li dendrites versus simulation time in the graded particle system.

Movie S5. Video of phase-field simulations for the self-limiting behavior of Li dendrites.
Lithium deposition at the interface between LZC and Li_3N.

Movie S6. Video of phase-field simulations for the self-limiting behavior of Li dendrites.
The variation of hydrostatic pressure at the interface between LZC and coarse Li_3N particles.

Movie S7. Video of phase-field simulations for the self-limiting behavior of Li dendrites.
The variation of hydrostatic pressure at the interface between LZC and Li_3N.
